# Variability in Recommendations for Cervical Lymph Node Pathology for Staging of Canine Oral Neoplasia: A Survey Study

**DOI:** 10.3389/fvets.2020.00506

**Published:** 2020-08-13

**Authors:** Michael Congiusta, Jessica Lawrence, Aaron Rendahl, Stephanie Goldschmidt

**Affiliations:** ^1^College of Veterinary Medicine, University of Minnesota, St. Paul, MN, United States; ^2^Masonic Cancer Center, University of Minnesota, Minneapolis, MN, United States; ^3^Department of Veterinary and Biomedical Sciences, University of Minnesota, St. Paul, MN, United States

**Keywords:** lymph node (LN), lymphadenectomy, oral tumor, staging, neoplasia, biologic behavior

## Abstract

There is no clear guideline regarding the indication for routine lymph node extirpation and pathologic evaluation during staging of canine oral tumors, despite a relatively high reported nodal metastatic rate for select tumor types. It is particularly unclear if clinicians recommend removal of lymph nodes only when there is confirmation of metastasis, defined as the N+ neck, or if elective neck dissection (END) is routinely recommended to confirm the true pathologic metastatic status of lymph nodes in the clinical N0 neck (no evidence of metastasis on clinical staging with diagnostic imaging or cytology). When clinicians are recommending END as a staging tool to confirm nodal status, there is also ambiguity regarding the surgical extent for subsequent histopathologic evaluation. The objective of this cross-sectional survey study was to determine the current recommendations given by practicing specialists regarding lymph node removal for dogs with oral tumors. Overall, 87 responses were obtained from 49 private practices (56%) and 38 academic institutions (44%). Respondents identified as oncologists (44%, *N* = 38), soft tissue surgeons (40%, *N* = 35), and dentists (16%, *N* = 14). Regardless of tumor type and stage, extirpation and histopathology were most commonly recommended in the clinical N+ neck only. The recommendation to routinely perform END in the N0 neck was significantly associated with tumor type. Bilateral removal of the mandibular and retropharyngeal lymph nodes was recommended more often for oral malignant melanoma (OMM) than for oral squamous cell carcinoma (OSCC; *p* ≤ 0.0039) or for oral fibrosarcoma (OFSA; *p* ≤ 0.0007). The likelihood of recommending END increased with increasing tumor size. Academic clinicians were significantly (*p* < 0.01) more likely to recommend END compared to private practitioners for canine T1–T3 OMM, T3 OSCC, T2 OFSA, and MCT. This study highlights the variability in recommendations for lymph node pathology for dogs with oral tumors. While tumor type and size influenced the decision to pursue END, it was not routinely recommended, even for tumor types with a known propensity for metastasis. Prospective studies are warranted to determine the potential diagnostic and therapeutic value of END in the N0 neck in veterinary patients such that a consensus approach can be made.

## Introduction

Tumor staging including the evaluation of locoregional draining lymph nodes for metastasis is a critical step in the oral oncologic work-up. For tumors of the head, presence of lymph node metastasis can affect treatment recommendations and may negatively impact outcome (1–7). Arguably, failure to properly identify the lack of lymph node involvement could also result in unnecessary treatment that fails to improve outcome. Lymphatic drainage from the head can be complicated making prediction of the most likely lymphocenter for metastasis challenging (3, 8–11). Furthermore, relying on lymph node size alone has been shown to be unreliable for accurate identification of metastatic disease, with palpably normal nodes commonly harboring metastasis (12, 13). While the mandibular, retropharyngeal and parotid lymphocenters represent the major draining sites for oral and maxillofacial tumors, the mandibular lymph nodes (MLN) are the most peripherally accessible for fine needle aspiration (FNA) and therefore the most commonly sampled (14). Not only may the tumor metastasize to nodes other than the MLN, but dogs can have multiple MLNs and it is not clear that all are evaluated during staging (3, 14). Importantly, assessment of only one MLN is unreliable at ruling out locoregional metastasis (8, 11). Similar to assessment of size, imaging alone is insufficient for prediction of metastasis, even with the use of computed tomography (CT), and is not recommended as a reliable tool without concurrent cytologic or histopathologic evaluation (15). Ultrasound or CT guidance may aid in the cytologic sampling of the less accessible retropharyngeal and parotid lymph nodes (RLN and PLN, respectively), depending on operator experience and comfort (10). Conversely, several veterinary oncologic studies have shown that cytology alone also lacks sensitivity and specificity in the evaluation of potential metastasis to regional lymph nodes (3, 16, 17). Thus, histopathologic evaluation of the draining regional lymph nodes at risk for metastasis remains the gold standard diagnostic staging tool for dogs with oral tumors (3, 8, 10, 11, 16, 17).

Similar concerns are noted for accurate detection of cervical metastatic disease in human oncology, with palpation, diagnostic imaging, and cytologic evaluation failing to replace elective neck dissection (END) for patients with the clinically negative (cN0) neck. The cN0 neck is defined as having no identified metastatic nodal disease with clinical staging utilizing palpation, diagnostic imaging, and/or cytologic sampling (18–20).

END by definition is removal of lymph nodes to confirm the true pathologic metastatic status in the clinical N0 neck. It may include ipsilateral or bilateral removal of the draining cervical lymphocenters, encompassing either a subset or all of the possible draining nodes. Conversely, therapeutic neck dissection refers to the removal of known metastatic lymph nodes (N+ neck), used to decrease tumor burden and improve discomfort associated with bulky nodal disease (21).

In early stage human head and neck tumors, where squamous cell carcinoma (SCC) is the predominant tumor type, an early recommendation was for END to be performed to confirm pathological node-negative disease (pN0) when the estimated risk of occult metastases from the tumor exceeds 20% (22). This risk analysis was based on the fact that pathologically detected occult metastases decreases 5 year survival by 50% (21, 23–26); undetected cervical metastasis therefore carries significant therapeutic and prognostic implications.

Human head and neck cancer data has identified tumor specific risk factors associated with cervical metastasis. Specifically, tumor location (tongue, floor of mouth), tumor size (>2 cm), and depth of invasion (>4 mm) have been repeatedly associated with an increased risk of nodal metastasis, leading to published guidelines outlining clear indications for END (21, 27–30). Additional data has shown that END reduces regional nodal recurrence and improves disease-specific survival for cN0 head and neck carcinoma, even for small tumors (20, 21, 23, 27, 30–35), and that up to 40% of patients without obvious nodal metastases at presentation will have occult metastases following dissection (36).

Critics of END in human oncology focus on the potential over-treatment argument and highlight that a large percentage of patients with clinically N0 neck assessment undergo unnecessary surgery, and it is not always clear that benefits outweigh the postoperative morbidity (21, 24, 26). Risks associated with END include prolonged anesthesia time, and postoperative changes including neck pain, fibrosis, reduced shoulder mobility and strength, and nerve damage, all of which can negatively affect patient quality-of-life measures (24, 32, 37, 38). As rapid advances in CT, MRI, and PET technology better detect nodal changes during cancer staging, it is possible that close observation with salvage neck dissection, defined as removing bulky metastatic disease at the time of nodal progression, may yield similar outcomes to END (24).

Sensitive methods for sentinel lymph node (SLN) mapping and biopsy, may also provide less invasive methods for some patients while still conferring the benefit of accurate staging with pathologic evaluation of the lymph nodes. SLN mapping involves identification of the first draining lymph node from the oral tumor, termed the SLN, and histopathologic evaluation in order to predict the status of the entire cervical region. SLN mapping techniques have been validated to accurately predict the entire lymphatic basin over 90% of the time, and have been shown to be a reliable staging tool for early stage human SCC (39–41). Studies directly comparing disease free survival time between SLN mapping with biopsy vs. END for the cN0 neck suggest SLN mapping may be a promising substitute in some circumstances to END (41–43).

Contrary to humans, tumor specific risk factors for cervical metastasis are not clearly defined in dogs; thus, it is difficult to understand if common guidelines lead clinicians to recommend END as a component of thorough staging protocol for oral tumors. Several recent studies have highlighted efficient and thorough methods for END that permit surgical staging for regional metastasis (10, 11, 44–46); but, a lack of clarity exists regarding which techniques clinicians are utilizing, and when they are recommending END, given the potential morbidity associated with this procedure. Possible surgical complications associated with END include seroma formation, infection at the lymphadenectomy site, and laceration of major vessels in the area, all of which could significantly impact the quality of life of the patient. Although, most complications are anecdotally reported as rare, the exact complication rates associated with END in dogs are not reported in the literature (10, 11, 44–46). Consideration also needs to be given to the additional surgical skill and time required to perform this procedure, as well as the associated cost to the client for additional surgery that may not result in a difference in overall survival time (46). Lastly, and potentially most impactful, the true prognostic effect of cervical metastasis on median survival time is unknown due to inconsistencies in both staging methodology as well as long term oncologic surveillance in canines. Thus, it is unclear at this time, if the benefit of END to confirm true pathologic status of the cervical lymph nodes will result in improved disease-free or overall survival, as it does in humans, where outcome is often evaluated in 3 or 5 year increments.

Standardization of approach for pathologic evaluation of cervical lymph nodes will minimize differences between studies regarding metastatic rate and outcome, thereby improving current knowledge to alter care paradigms and allowing evaluation of tumor-specific risk factors for cervical metastasis. Therefore, the purpose of this study was to assess current practice amongst veterinary specialists who routinely stage and treat canine oral tumors. The goal was to determine the variability in staging and therapeutic recommendations currently made for common canine oral tumors. We hypothesized that specialists would recommend END in oral malignant melanoma (OMM) and large (>4 cm) oral squamous cell carcinoma (OSCC) more frequently than for other tumors, based on the current body of veterinary literature on metastatic risks.

## Materials and Methods

### Survey Questions

A link to an electronic questionnaire formulated in Microsoft Outlook (available in Appendix 1) was provided to members through distribution via the email list server (listserv) for the American Veterinary Dental College (AVDC), the Veterinary Society of Surgical Oncology (VSSO), the American College of Veterinary Surgeons (ACVS), the American College of Veterinary Internal Medicine-Oncology (ACVIM-Oncology), and the American College of Veterinary Radiology-Radiation Oncology (ACVR-Radiation Oncology). The survey was accessible for a 4 month duration, and three reminders were emailed to request completion. This study was exempt from review by the University of Minnesota Institutional Review Board (IRB).

The questionnaire requested that respondents follow guidelines (Table 1) and make particular assumptions prior to taking the survey to permit standardized results. Tumor types queried with respect to clinical staging were limited to OMM, OSCC, and oral fibrosarcoma (OFSA). Questions regarding tumor stage utilized the World Health Organization staging scheme describing the primary tumor (T), status of regional lymph nodes (N) and presence or absence of distant metastasis (M) (47). Specifically, T1 referred to tumors ≤ 2 cm in diameter, T2 referred to tumors 2–4 cm in diameter and T3 referred to tumors >4 cm in diameter (47).

**Table 1:** Lymph node recommendation for tumor type and stage: guidelines when answering the survey.

1.	Please indicate lymph node recommendations for the following oral neoplasms using the WHO TNM Classification scheme
2.	For all staging questions regarding tumor size (T1–T3), assume there is no evidence of distant metastasis
3.	For all patients, assume no patient has a concurrent comorbidity and that each client is not concerned about finances to provide optimal treatments to best manage clinical symptoms of their animal
4.	While there is no clear consensus statement on proper lymphadenectomy procedure guidelines, please provide answers that fit with your strongest recommendation

OSCC, OMM, OFSA was queried separately for T1–T3 as well as stage 4. All questions pertaining to the T1–T3 tumor types had the same 5 multiple choice options to choose from Table 2. For stage 4 tumors, defined as presence of distant metastasis, respondents were also provided an alternative option regarding lymph node extirpation to clarify if they recommend removal if it aligns with the client goals and/or improves quality of life of the patient. For stage 4 tumors, it was assumed that distant metastasis was confirmed with diagnostic imaging, although this was not clearly defined within the survey.

**Table 2:** List of possible survey responses.

1	All	Recommend removing retropharyngeal, mandibular, and parotid lymph nodes bilaterally regardless of normal appearance on diagnostic imaging
2	All	Recommend removing retropharyngeal and mandibular lymph nodes bilaterally regardless of normal appearance on diagnostic testing
3	All	Recommend removing retropharyngeal and mandibular lymph nodes ipsilaterally regardless of normal appearance on diagnostic imaging
4	All	Recommend removing a regional lymph node only if it is suspicious on diagnostic imaging and/or suspicious/positive for metastasis following cytologic assessment
5	All	Other
6	Stage 4 only	Recommend removing the regional lymph node(s) depending on the goals of the client and on the clinical signs and/or quality of life of the pet (i.e., palliative radiation therapy to primary tumor/regional LN and/or medical management may be elected in lieu of surgery)

*Responses were listed as multiple choice options (1-5) that pertained to all tumor questions. For stage 4 dogs, respondents were also provided with an additional option (6)*.

Within the survey, END was defined as either bilateral removal of RLN, MLN, and PLN, bilateral removal of RLN and MLN, or ipsilateral removal of MLN and RLN when there was no evidence of lymph node metastasis on cytology or diagnostic imaging (cN0). Conversely, removal of a single node or nodes based on high suspicion or confirmation of metastasis was considered to be therapeutic, not elective, neck dissection.

Lymph node management for uncommon tumor types were queried via additional questions without regard to tumor size. Uncommon tumor types queried included osteosarcoma, chondrosarcoma, and mast cell tumor (MCT). For osteosarcoma and chondrosarcoma, respondents were queried yes or no if their recommendations are the same for both these tumors as they are for OFSA. For MCT respondents were given the same 5 multiple choice options as common oral tumors (Table 2).

To gather information about alternative methods of cervical node management, the survey also asked respondents if SLN mapping was routinely performed for head and neck tumors and to define the protocol if one existed. This section on SLN mapping was free text to allow clinicians to specify the protocol used to identify the first draining lymph node from the oral tumor, which is presumed to be the sentinel lymph node (although the use of SLN biopsy to predict the metastatic status of the entire basin has not been validated in canines). Respondents were also asked to subjectively report how often the mapping technique accurately identified the first draining lymph node.

Additional information regarding subjective complications following END was also recorded. Specifically, respondents were asked to report approximate occurrence of postoperative seroma as stratified answers of: often (>75% of the time), frequent (50% of the time), rarely (<25% of the time), or other. Respondents were also queried about postoperative infection and given the same possible responses as for seroma.

### Statistical Analysis

To assess overall differences in response by practice type (academia vs. private practice) and specialty, Fisher's test was performed using the algorithm in the fisher.test R function. This was done separately for each question. To follow-up statistically significant differences, pairwise Fisher's tests were performed, for each response separately against all others. For specialty, this was done pairwise between the three specialties, with the Bonferroni-Holm adjustment for multiple comparisons. Adjustment, however, was not performed across the possible different answers for each question. First, because the overall Fisher's test controls the overall Type I error, and second, because the responses are necessarily correlated (that is, if there are more of one response, there must be less of another). To assess overall differences in response between the three common tumor types (OMM, OSCC, and OFSA), a different method was needed, because these were repeated responses from the same individuals. Therefore, a permutation-based chi-squared test was used, with permutations formed by permuting the three responses within each respondent. This was done separately for (T1–T3), and 20,000 permutations were performed for each. To follow up statistically significant differences, pairwise McNemar's tests were performed, with *p*-values computed using the exact binomial test. Adjustment for multiple comparisons was performed similarly to the adjustments for the specialties. Lastly, for each possible response separately, pairwise McNemar's tests was performed between all 11 groups (T1, T2, T3 OMM, SCC, FSA as well as mast cell tumor and lymphoma) with *p*-values corrected using the Bonferroni-Holm method. For each of the 11, significant difference between responses was defined at the *p* < 0.05 level. This was repeated with responses 1–3 combined to represent recommendation for any form of END. All computations were performed with standard statistical software.^1^

## Results

Overall, 87 surveys were completed. Fifty-six percent (49/87) of the responses were from private practices and 43% (38/87) were from academic institutions. None of the survey responses were excluded from analysis. However, information on lymph node management for epitheliotrophic lymphoma as well as one survey question on SLN mapping were removed from analysis due to ambiguous wording and inconsistent responses (Appendix 1). Respondents identified as practicing oncology (44%, *N* = 38), soft tissue surgery (40%, *N* = 35), or dentistry and oral surgery (16%, *N* = 14). No respondent identified as a non-boarded specialist when queried on their specific specialty in the survey.

### Lymph Node Recommendations for Common Oral Tumors

**Figure 1:**
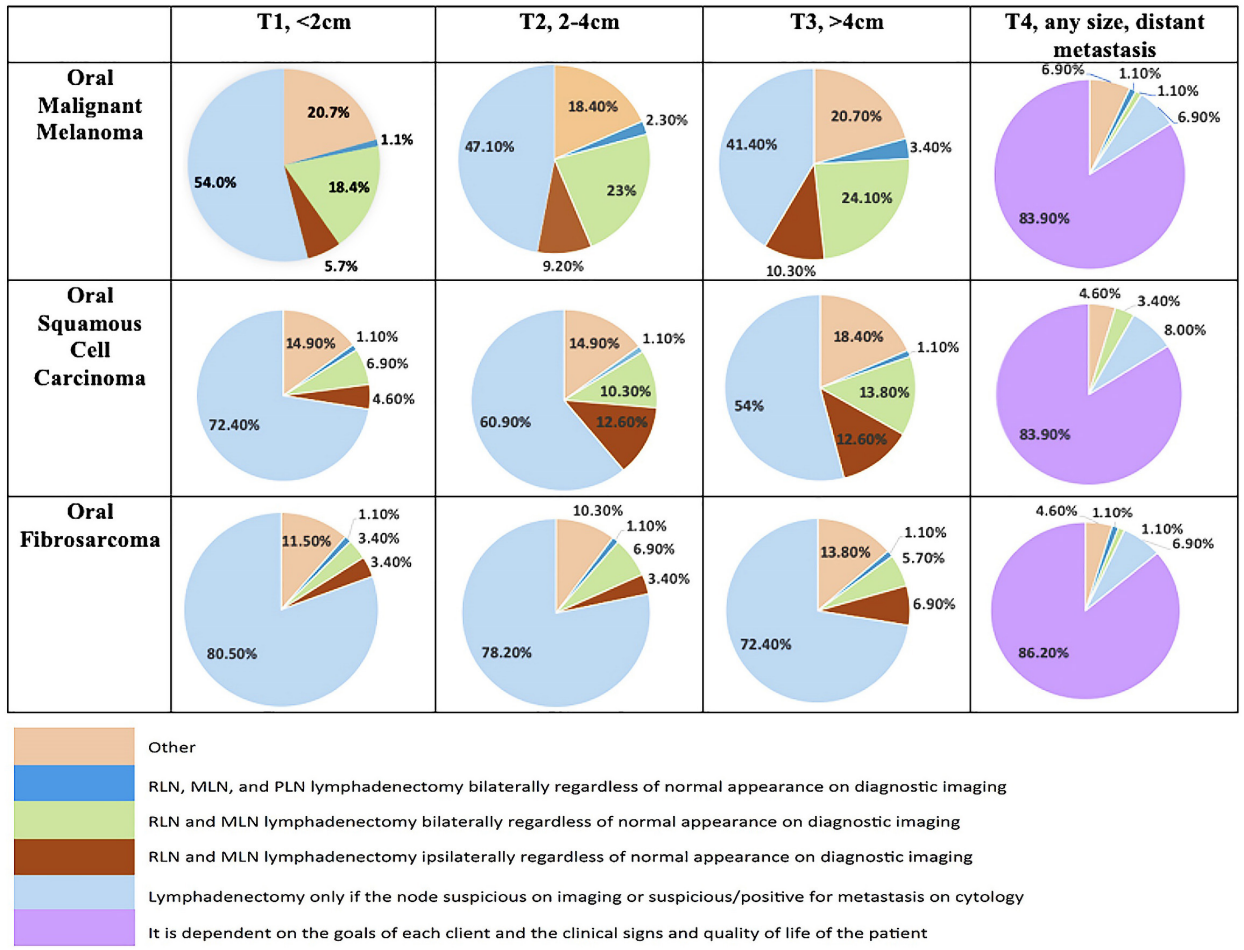
All survey responses for clinician recommendations regarding performing lymph node pathology for common tumor types separated by stage.

**Figure 2:**
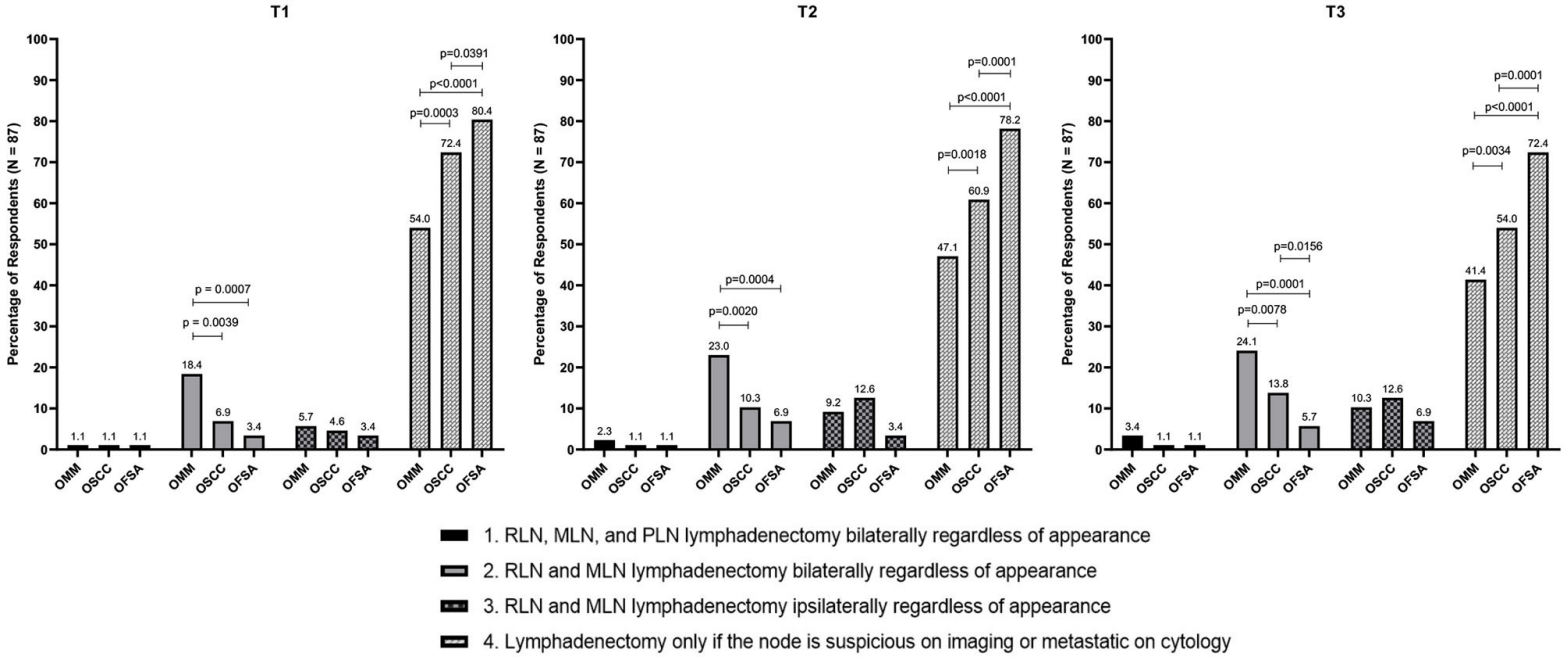
Recommendation to perform END in the N0 neck vs. extirpation in the N+ neck only for T1–T3 OMM, OSCC, OFSA. Significant differences between specific recommendations per tumor type are highlighted.

Clinicians most commonly recommend lymph node extirpation for histopathologic analysis only when there is clinical suspicion of metastasis (N+ neck), regardless of tumor type (Figure 1). Accordingly, the majority of clinicians do not routinely recommend END (Table 3) for cN0 oral tumors in dogs. When END is recommended, it is recommended more frequently for OMM compared to OSCC and OFSA for similar clinical stages (Figure 2).

**Table 3:** Overall recommendations for any form of elective neck dissection (END) for common canine oral tumors (OMM, OSCC, OFSA) for clinically neck negative (N0) disease.

**OMM**			**OSCC**			**OFSA**		
**T1**	**T2**	**T3**	**T1**	**T2**	**T3**	**T1**	**T2**	**T3**
22 (25.2%)	30 (34.5%)	33 (37.9%)	11^*^ (12.6%)	21 (24.1%)	24^*^ (27.6%)	7 (8.0%)	10 (11.5%)	12 (13.8%)

*In this study, any form of END included bilateral removal of RLN, MLN, and PLN, bilateral removal of RLN and MLN, or ipsilateral removal of MLN and RLN. Data are presented as the number of respondents who elected END with the percentage of respondents in parentheses*.

* *Significant (p < 0.01) difference between T1 and T3 OSCC*.

Within this study population it was identified that when END is recommended, parotid lymph node extirpation is rarely included (Figure 2). Bilateral removal of RLN and MLN is more commonly recommended for OMM, while ipsilateral or bilateral extirpation is recommended with similar frequency for OSCC and OFSA. Notably, bilateral extirpation of the MLN and RLN in the cN0 neck is recommended significantly more frequently for OMM than for OSCC and OFSA, and more often (*p* < 0.01) for T3 OSCC than for T3 OFSA (Figure 2). Selective extirpation of only a suspicious or confirmed metastatic node is performed significantly more frequently (*p* < 0.05) for OFSA compared to OSCC and OMM, as well as OSCC compared to OMM (*p* < 0.01) (Figure 2).

As tumor size increased, regardless of tumor type, clinicians recommend END more often, although this difference was often not significant (Table 3). No significant difference was identified in the likelihood of END recommendation for dogs with OMM or OFSA with increasing tumor size. For OSCC, clinicians are significantly (*p* < 0.05) more likely to recommend END for T3 tumors compared to T1 tumors.

Regardless of tumor type, once there is evidence of distant metastatic spread, END is only recommended in 2–4% of cases (2.2% OMM, 3.4% OSCC, 2.2% OFSA) (Figure 1).

### Lymph Node Recommendations for Less Common Oral Tumor Types

#### Osteosarcoma and Chondrosarcoma

When queried as a yes or no response, 77% of respondents replied that they make the same recommendations for both osteosarcoma and chondrosarcoma (67/87) regarding lymph node extirpation for histopathologic analysis as they do for OFSA.

#### Oral/Labial Mast Cell Tumor (MCT)

**Figure 3:**
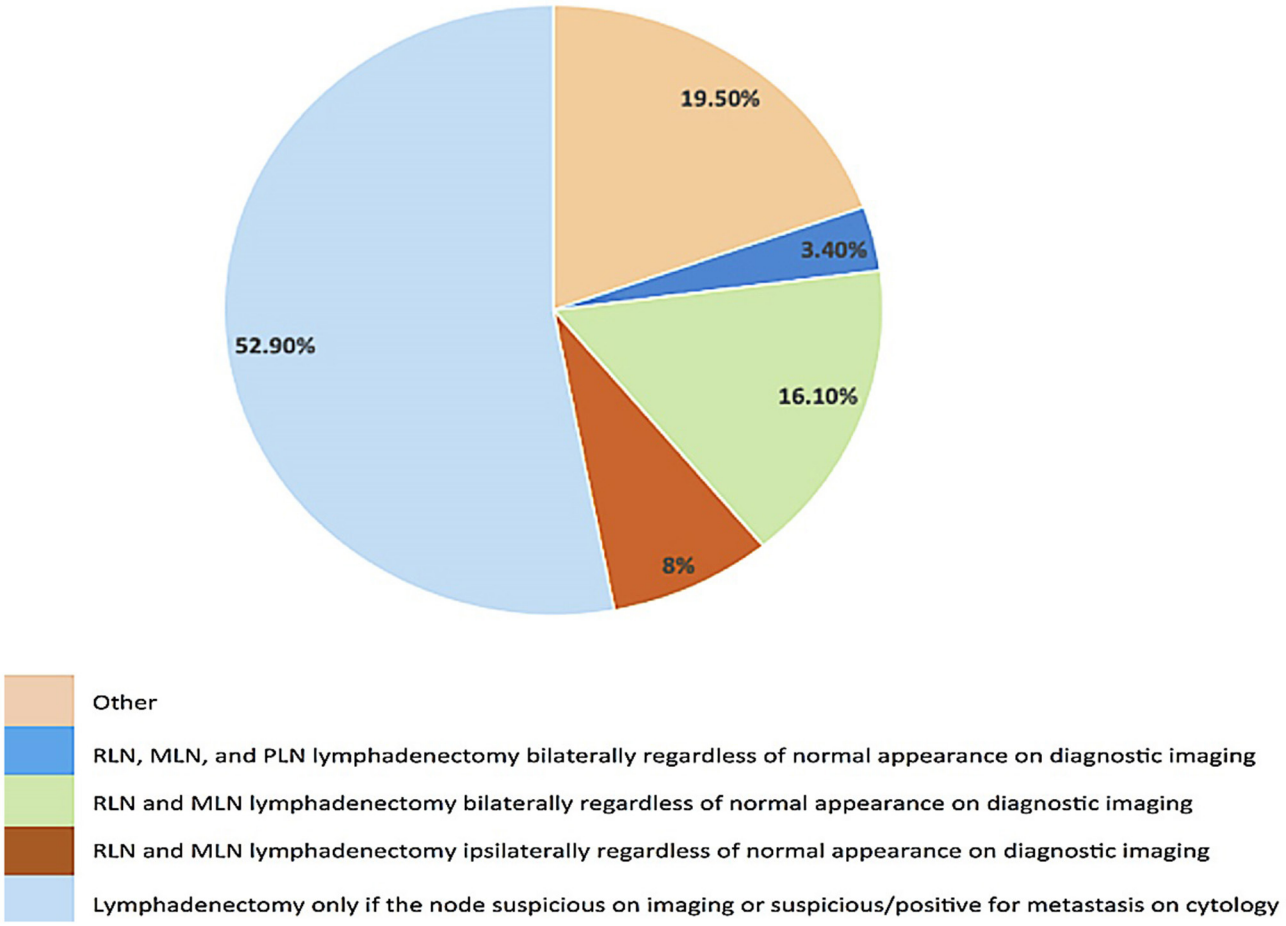
Survey responses for clinician recommendations regarding performing lymph node pathology for oral/labial mast cell tumor.

Twenty seven percent of clinicians responded that END is recommended for dogs with oral MCT (Figure 3). There was no significant difference in the frequency of some form of END recommendation in dogs with MCT compared to T1–T3 OMM, T2–T3 OSCC, and T3 OFSA. When END is recommended, bilateral removal of the MLN and RLN is the most common (58.3%) surgical recommendation.

### Use of SLN Mapping

Thirty-three percent (29/87) of respondents reported use of SLN mapping techniques for head and neck tumors. Twenty- one respondents reported the exact technique utilized, with some reporting more than one technique that is utilized at their institution. Reported techniques included CT lymphangiography +/– methylene blue at surgery (19/21), intra-operative use of methylene blue alone (1/21), lymphoscintigraphy (3/21), and intraoperative near infrared fluorescence lymphography (4/21). Of those that responded that they perform SLN mapping, the majority (66.7%) subjectively reported they are able to accurately identify the first draining LN (presumed SLN) >50% of the time. Specifically, 23.3% of respondents claimed a 50–75% success rate, and 43.3% reported a 75–100% success rate using their respective mapping protocols. Only 8 respondents reported they request additional pathologic information on the removed “sentinel lymph node.” Reported written requests included: breadloafing (2/8) immunohistochemistry (2/8), polymerase chain reaction (1/8), and bivalving the SLN through the hilum (2/8). Kiupel mast cell tumor staging (1/8) was also listed, however due to the anonymous nature of the survey, the authors were unable to determine if this was meant to reflect a request for grading of the primary tumor, or if the respondent uses a unique lymph node evaluation method with criteria similar to the Kiupel grading system (48).

### Complications With Elective Lymphadenectomy

The self-reported complication rate of seroma formation following END was low, with 6.9% reporting it is never seen and 47.1% reporting it is seen rarely (<25% of the time). The remainder reported it was seen ~50% of the time (19.5%), >75% of the time (16.1%), 100% of the time (3.4%) or reported the question was not applicable to them.

The self-reported infection rate following END was also low with 24.1% reporting this complication was never seen and 56.3% reporting it is seen rarely (<25% of the time). No respondents reporting that the complication was always or commonly (>75%) seen. The remainder reported that it was seen 50% of the time (2.3%) or that the question was not applicable to them.

Less common complications reported included facial swelling, regional edema, dehiscence, hemorrhage, and muzzle edema. Respondents were not queried on if they saw complications more commonly with specific forms of END compared to other surgical techniques.

### Recommendation Differences Based on Practice Type or Specialty

Clinicians in academia were significantly (*p* < 0.01) more likely to recommend routine bilateral END (RLN and MLN) for T1–T3 OMM, T3 OSCC, T2 OFSA, and oral MCT compared to clinicians in private practice. Conversely, clinicians in private practice were significantly (*p* < 0.01) more likely to make recommendations for solitary lymph node extirpation based on suspicion of metastasis. Academic clinicians were significantly (*p* < 0.01) more likely to perform SLN mapping compared to private practices. No significant differences in recommendations between specialties were found for dogs with OMM, OSCC, OFSA, or oral mast cell tumors.

## Discussion

This is the first study to investigate current practice for the management of regional lymph nodes in dogs with oral tumors. There is no current consensus for lymph node management or neck dissection in the clinically negative (cN0) neck or in the metastasis-positive or suspected positive (N+) neck. The lack of standardized approach among board certified small animal oncologists, dentists, and surgeons to the determination of lymph node status prohibits the realization of the true prevalence of metastasis for canine oral tumors and evaluation of tumor specific risk factors for cervical metastasis. This study therefore sought to determine recommendations provided by specialty clinicians who routinely stage and treat canine oral tumors.

It was identified that clinicians are most likely to recommend bilateral END for OMM compared to other tumor types. However, a large proportion of clinicians (41–80% of respondents pending tumor type and stage) elect to remove only a suspicious or cytologically confirmed metastatic lymph node for dogs with oral tumors.

This finding was not surprising for canine OFSA given the low likelihood of lymph node metastasis (6, 49–51); indeed, even for T2 and T3 tumors, clinicians only recommend END in 11 and 14% of cases, respectively. Furthermore, the true prognostic effect of cervical metastasis with OFSA is elusive due to limited data. In one study of OFSA, only 3% of dogs had lymph node metastasis at diagnosis and it was not associated with outcome (51). A second study of OFSA in 29 dogs did not detect any nodal metastasis at diagnosis while local recurrence and distant metastasis were the documented causes of death (50). Overall, the risks of node extirpation unlikely outweigh any potential benefits. Similar to canine OFSA, respondents largely reported similar lymph node management for uncommon oral sarcomas such as osteosarcoma or chondrosarcoma.

Unexpectedly, only 25–38% of clinicians recommended END in the N0 neck for dogs with OMM, despite the high (up to 74%) propensity of these tumors to metastasize to regional lymph nodes, often to multiple lymphocenters (7–9, 11, 13, 17, 52). This was especially surprising given that existing literature suggests that lymph node size, imaging appearance, and cytologic evaluation are limited in accurately determining nodal metastasis for canine OMM (3, 8, 9, 13, 15, 17). It is possible that respondents have factored in several findings in their clinical management strategies, such as: the uncertainty in the true rate of cervical lymph node metastasis in canine OMM in part due to lack of standardized assessment, that the literature has not always shown lymph node metastasis to be a negative prognostic factor, and that distant metastasis is particularly problematic for many cases (7, 53–56). The risk of concurrent distant metastasis may also explain why END was not increasingly performed with advancing tumor size for OMM. It is well-documented that as tumor size increases, the risk of distant metastasis concurrently increases; thus, even with the lack of confirmed metastatic deposits in the thorax, the morbidity of performing lymphadenectomy may not be justified given the potential for decreased median survival time (7, 52). This is a controversy that surrounds human melanoma as well and forms the basis of the primary argument against END for staging human head and neck cutaneous melanoma. This argument stems around the “marker hypothesis,” which supposes that the presence of metastatic deposits in the cervical lymph node(s) act only as a marker for distant dissemination and removal of the tumor burden in the nodal bed carries no therapeutic value itself (57, 58). Thus, END used solely for staging and prognostic purposes, without a positive effect on survival, cannot be justified in the face of its associated morbidity; this may be particularly relevant when considering tumors like metastatic melanoma that are challenging to treat systemically (57, 58).

Recommendations made for OSCC were in accord with the majority of available veterinary literature suggesting overall low rates (9–13%) of nodal metastasis at diagnosis (2, 6, 8, 10) and increasing risk of metastasis with larger tumors (5, 59, 60). Notably, clinicians were significantly more likely to recommend END for T3 tumors compared to T1 tumors. Although the recommendation for END increased as tumor size increased, within each T stage, clinicians were still more likely to recommend bilateral removal of the MLN and RLN for dogs with OMM compared to OSCC. This recommendation mirrors the reported biologic behavior of these two tumor types. However, when looking at a recommendation for any form of END (not just bilateral MLN and RLN removal), there was no significant difference in the likelihood to recommend END for a T2/T3 OSCC or OMM. This suggests that clinicians feel that larger OSCC may have a higher nodal metastatic rate. Indeed, a recent study did not identify significantly different percentages of cervical metastasis in dogs with OMM and OSCC following histopathologic evaluation (8). Notably, the majority of dogs with OSCC in that study had T2 or T3 tumors but none had suspicion of nodal metastasis with staging; however dogs with tonsillar SCC were also included in the study cohort, which impacted the reported metastatic rate (8). In a separate case series that studied efficacy of piroxicam and carboplatin in dogs with T3 non-tonsillar OSCC, all dogs had nodal metastasis (61). The identification of nodal metastasis is clinically significant for OSCC, as literature has reported that median survival time is reduced by 67–90% when nodal metastasis is present (1, 6). Furthermore, unlike melanoma, there is stronger evidence to suggest that carcinoma stalls in the lymphatic channels prior to hematogenous dissemination, which supports that lymphadenectomy may be therapeutic in addition to prognostic (21, 34). Recent literature surrounding T2–T3 OSCC in canines suggests that routine END might be justified, although its prognostic and therapeutic value requires further investigation.

Interestingly, despite the paucity of literature on oral MCTs (4, 62), respondents were equally as likely to recommend END (27.5%) for dogs with oral/labial MCTs as they were for OMM (approximately 32.5% overall). Unlike OMM, where distant metastasis poses a significant clinical challenge (5, 7, 52, 63), literature supports that adequate local control of the primary tumor and regional nodes is the most important facet for long-term control for dogs with MCTs (54–56). Thus, it was surprising that given the high propensity for oral MCT to metastasize to lymph nodes and the significant effect of nodal metastasis on median survival time, that END is not recommend more often for the N0 neck (54–56). Rather, in 53% of cases, nodes are extirpated only if suspicious for metastasis on imaging or cytologically confirmed as metastatic.

Overall regardless of tumor type, clinicians are most likely to recommend lymph node extirpation only if there is clinical suspicion or cytologic confirmation of metastasis. The survey was not designed to assess subsequent clinical decision making, so it could not accurately capture the frequency at which recommendations changed upon clinical staging, or what served as rationale behind this recommendation. Specifically, the survey asked that respondents choose between recommending removal of a lymph node only where there is suspicion of metastasis (N+) or various forms of END in the cN0 neck (or other); it was therefore challenging to capture clinical cases where, following identification of a suspicious lymph node during staging, END was performed to remove other cervical nodes at risk. Furthermore, the survey was not designed to determine if radiotherapy was planned in order to achieve local control following identification or confirmation of a suspicious lymph node, rather than surgical extirpation.

What was clear from the survey results, however, is that END is not routinely recommended in the cN0 neck for dogs with oral tumors. This may be due to the morbidity associated with lymphadenectomy without a clearly defined benefit in survival times, or a lack in understanding surrounding the importance of examining lymphocenters other than the MLN (8, 11).

Of the subset of respondents that routinely recommend END, the majority recommend bilateral removal of the RLN and MLN for OMM, while bilateral and ipsilateral removal of MLN and RLN is recommended with similar frequency for OSCC and OFSA. The canine literature points out the risk of lymph drainage in the head occurring on the contralateral side in up to 62% of cases (8, 10, 11). Thus, for complete evaluation of cervical metastasis, removal of the lymph nodes bilaterally should be performed especially if the tumor is at midline (8, 9, 45). There may be a propensity for tumors at certain locations to spread only ipsilaterally, but further evaluation of metastatic lymphatic patterns in the dog are required to identify when ipsilateral lymphadenectomy may be more appropriate. Until more robust data on lymph node metastatic drainage patterns is available, it seems prudent to perform bilateral RLN and MLN for high risk oral tumors (9, 10, 45).

It is also important to note, that dogs can have differing numbers of mandibular nodes (range of 2–4 is most common) as well as both a medial and lateral retropharyngeal lymph node (14). The survey was not designed to specifically question how clinicians manage aberrant anatomy. Ideally, CT scan with IV contrast should be performed prior to END to highlight aberrant anatomy and guide surgical planning. Failing to remove all possible draining lymph nodes for pathologic evaluation may hinder END performance in the cN0 neck, as the first draining node of the lymphatic basin may not be pathologically evaluated. In the future, the use of SLN mapping techniques may help highlight which node(s) are most important to sample for staging purposes.

Of respondents performing END, the self-reported complication rate was low, with the majority reporting infection and seroma as potential complications. While the survey was not designed to address specific complications and severity, in retrospect, a critical flaw was that the survey did not differentiate transient cervical edema from true seroma formation. This potentially overestimates the reported frequency of seroma formation and underestimates cervical edema formation in our study. Furthermore, the questions surrounding complications also queried oncologists, which may not be an accurate reflection of the complication rate as they most likely are not overseeing the follow up for these patients postoperatively. Additional study is needed to characterize adverse events associated with END procedures, particularly as seroma and postoperative infection cause discomfort and may delay adjuvant therapy in some dogs. For example, postoperative seroma may delay the start of treatment or require adaptive planning (re-planning due to tissue changes in the radiation field) (64, 65). Decreases in seroma volume or resolution of a postoperative abscess after CT simulation for radiation planning and/or during treatment negatively affect the quality of the radiation plan and may significantly increase the volume of and dose to normal tissue if uncorrected, thus increasing the likelihood that adverse events will develop (65, 66). While adaptive planning techniques are routine in some human radiation oncology departments, the use of adaptive planning in veterinary oncology is not yet standard and carries financial and time commitment implications for pet owners.

Given the infrequency in which END is currently recommended, and the potential for morbidity with neck dissection, it raises the question of whether or not the potential benefit of END is worth the associated morbidity in the canine N0 neck.

This same argument exists in human literature, where the true necessity of END still remains uncertain for early stage disease OSCC, despite updated meta-analyses and guidelines (21, 22, 24, 32, 36, 67). In favor of END is literature that has shown that occult metastases can be detected in 10–45% of cases despite a N0 clinical assessment and the fact that the presence of nodal metastasis carries clear prognostic implications decreasing 5 year survival by up to 50% (21, 23–26). Based on this, it has been historically recommended that END is performed when the cervical metastatic risk of the tumor exceeds 20%. However, in opposition to END is the fact that this means that up to 80% of patients are receiving unnecessary neck dissection, which can be associated with significant morbidity. Due to the controversy surrounding END, SLN mapping, and biopsy was introduced and is regarded as an alternative staging method to END with similar long-term survival outcomes (41).

In this study, 30% of respondents reported that they routinely recommend and perform SLN mapping techniques in canines to identify the first draining lymph node for oral tumors. This low percent was unsurprising since SLN techniques have not been standardized or validated in veterinary medicine. While a comprehensive review of SLN techniques is beyond the scope of this study, the reader can be directed toward a recent review of promising methodologies (68). Briefly, SLN mapping techniques primarily include peritumoral injection of either iodinated contrast, methylene blue dye, or radioactive isotopes and then evaluation of which node the injected material drains to first, utilizing either preoperative diagnostic imaging, a gamma probe, or visual detection surgically. This first draining lymph node is referred to as the SLN, which has the potential to predict the status of all other nodes in the basin; thus, negating the need for END for confirmation of pathologic N0 status. In canines, several mapping techniques to identify the first draining lymph node in the neck have been reported (68–70). However, in dogs, it is not yet clear if pathology within the first draining lymph node accurately reflects the remainder of the lymphatic basin so further validation is needed for SLN mapping to replace END to confirm pN0 status.

One limitation of the survey study is that it did not clearly define what was meant by SLN mapping nor “success” of the SLN mapping procedure. Of those that responded they perform SLN mapping, 67% stated that they accurately identify the SLN the majority of the time, but it wasn't clear if success was defined by histopathologic assessment of metastasis as the gold standard. One key component to validating novel SLN mapping techniques requires histopathologic confirmation that the “sentinel node” identified accurately predicts the remaining lymph node basin, and thus can reliably be used in place of END. It is therefore essential to capture the false negative rate of SLN biopsy to validate the technique. This supports the establishment of recommendations for END as the gold standard for canine oral tumors at moderate to high risk of metastasis until validation of SLN techniques has been performed (71). Surprisingly, for those respondents who currently recommend or perform SLN mapping and biopsy, only 6% specify how they would like nodes to be evaluated histologically. One of the primary benefits of SLN mapping is that if only one or two nodes are submitted, more rigorous pathologic evaluation may be feasible. For example, thin serial sectioning of each node with immunohistochemistry (such as AE1/AE3) has been shown to significantly increase detection of occult metastases in humans (72–75), and these techniques may also be beneficial in canine tumors.

At our institution, we perform SLN mapping with CT lymphangiography followed by END (bilateral MLN and RLN) for tumors with a reported cervical metastatic risk of 20% or greater based on the current body of veterinary literature (2, 5, 7–11, 13, 17, 52, 59, 60), similar to early recommendations in human OSCC (22). Currently, we perform SLN mapping and END for T1–T3 OMM (excluding well-differentiated T1 melanomas with a mitotic index of 0–1), T2–T3 OSCC, and mucosal/oral mast cell tumors. However, the authors do acknowledge that the true cervical metastatic rate is not clearly defined and the treatment paradigm we have instituted will be updated as new literature is available.

It is our hope that with more data on the true metastatic risk of canine oral tumors and the prognostic effect of cervical metastasis, a more appropriate risk analysis and guideline for when to perform END could be adopted. By corroborating cervical metastatic status with factors such as tumor size, location, and pathologic features such as depth of invasion, mitotic index, and perineural invasion, specific risk factors may highlight dogs with oral tumors that most benefit from END approaches.

It is important to note that risk analysis comparing the benefit vs. risk of recommending routine END cannot be accurately performed within the canine population without knowledge of the true cervical metastatic rate of oral tumors as well as the prognostic implication of cervical metastasis. Further work needs to be done to characterize the rate of occult metastasis in the canine clinical N0 neck, document the rate of complications following END procedures, compare progression-free survival between END and observation or nodal irradiation, and determine the clinical case scenarios that unequivocally benefit from END.

As detailed, there were several limitations in the survey study, including inherent bias with self-reporting, low response rate which may have selected for clinicians with strong opinions for or against END, lack of definition or clarification of individual postoperative complications with END, lack of definition of a “successful” SLN mapping procedure, and the inability for respondents to rationalize their responses. However, it provides the first study to highlight the variability that exists across clinicians who stage and treat canine oral tumors. The recognition of this variability will hopefully encourage thoughtful protocols that improve our clinical decision-making.

## Conclusions

This is the first study to describe variability in lymph node management across veterinary specialty practices that routinely stage and treat canine oral tumors. Current practice is such that the majority of specialists who stage and treat canine oral tumors remove one or several cervical lymph node(s) for histopathologic evaluation based on clinical suspicion of metastases (i.e., pathology only performed in the N+ neck). For the cN0 neck, observation is more commonly recommended than END, although select practitioners recommend END for T2 and T3 canine OMM and OSCC, as well as mucosal MCT, consistent with their known higher risk of local metastases.

## Data Availability Statement

The datasets generated for this study are available on request to the corresponding author.

## Author Contributions

MC, JL, and SG: study concept and design, contributed to manuscript revision, and approved the submitted version. MC: data acquisition and initial draft of manuscript. AR: statistical analysis. All authors contributed to the article and approved the submitted version.

## Conflict of Interest

The authors declare that the research was conducted in the absence of any commercial or financial relationships that could be construed as a potential conflict of interest.

## Footnotes

^1^R Core Team. *R: A Language and Environment for Statistical Computing*. R Foundation for Statistical Computing, Vienna (2019). Available online at: https://www.R-project.org/.

## Abbreviations

SLN: Sentinel Lymph Node
RLN: Retropharyngeal Lymph Node
MLN: Mandibular Lymph Node
PLN: Parotid Lymph Node
END: Elective Neck Dissection. In this survey END included bilateral removal of RLN, MLN, and PLN, bilateral removal of RLN and MLN, or ipsilateral removal of MLN and RLN
cN0 neck: Cervical lymph nodes have staged clean with clinical assessment performed by palpation, diagnostic imaging, and/or cytology
pN0 neck: Cervical lymph nodes are confirmed to not have metastatic deposits by pathologic evaluation
N+ neck: Cervical lymph nodes have been shown to be metastatic or are highly likely metastatic based on clinical assessment with palpation, diagnostic imaging, and/or cytology
OMM: Oral Malignant Melanoma
OSCC: Oral Squamous Cell Carcinoma
OFSA: Oral Fibrosarcoma
MCT: Mast Cell Tumor.
