# Supplementary material for: Variability in Recommendations for Cervical Lymph Node Pathology for Staging of Canine Oral Neoplasia: A Survey Study
Source: Front Vet Sci. 2020 Aug 13;7:506. doi: 10.3389/fvets.2020.00506 (PMC7438545; doi:10.3389/fvets.2020.00506)
Supplement: Supplementary file 1 [file Data_Sheet_1.pdf]

Appendix 1: Survey questions

Lymph node recommendation for common oral tumors

Respondents were asked to indicate their lymph node recommendations for the following common oral neoplasms using the WHO TNM Classification scheme:

1.T1 Oral Malignant Melanoma (OMM), <2cm

2.T2 Oral Malignant Melanoma (OMM), 2-4cm

3.T3 Oral Malignant Melanoma (OMM), >4cm

4.T4 Oral Malignant Melanoma (OMM), any size, distant metastasis

5.T1 Oral Squamous Cell Carcinoma (OSCC), <2cm

6.T2 Oral Squamous Cell Carcinoma (OSCC), 2-4cm

7.T3 Oral Squamous Cell Carcinoma (OSCC), >4cm

8.T4 Oral Squamous Cell Carcinoma (OSCC), any size, distant metastasis

9. T1 Oral Fibrosarcoma (OFSA), <2cm

10. T2 Oral Fibrosarcoma (OFSA), 2-4cm

11. T3 Oral Fibrosarcoma (OFSA), >4cm

12. T4 Oral Fibrosarcoma (OFSA), any size, distant metastasis

The same responses to each question were made available.

1	All	Recommend removing retropharyngeal, mandibular, and parotid lymph nodes bilaterally regardless of normal appearance on diagnostic imaging.
2	All	Recommend removing retropharyngeal and mandibular lymph nodes bilaterally regardless of normal appearance on diagnostic testing.
3	All	Recommend removing retropharyngeal and mandibular lymph nodes ipsilaterally regardless of normal appearance on diagnostic imaging.
4	All	Recommend removing a regional lymph node only if it is suspicious on diagnostic imaging and/or suspicious/positive for metastasis following cytologic assessment.
5	All	Other.
6	Stage 4 only	Recommend removing the regional lymph node(s) depending on the goals of the client and on the clinical signs and/or quality of life of the pet (i.e. palliative radiation therapy to primary tumor/regional LN and/or medical management may be elected in lieu of

		surgery).

Lymph node recommendation for uncommon oral tumors

13. Do you recommend the same lymph node guidelines for chondrosarcoma and osteosarcoma in the oral cavity as you do for fibrosarcoma?

A. Yes
B. No
C. Other

Respondents were queried on the following round cell tumors and asked to assume dogs with round cell tumors have staged negative after assessment of distant metastasis

14. Oral Epitheliotropic Lymphoma, any size **** This question was removed from analysis and presentation in the results section due to confusion regarding the wording explaining that lymphoma was confined to the oral cavity.**

15. Oral/ labial Mast Cell Tumor, any size

The same responses for both tumors were made available

1	All	Recommend removing retropharyngeal, mandibular, and parotid lymph nodes bilaterally regardless of normal appearance on diagnostic imaging.
2	All	Recommend removing retropharyngeal and mandibular lymph nodes bilaterally regardless of normal appearance on diagnostic testing.
3	All	Recommend removing retropharyngeal and mandibular lymph nodes ipsilaterally regardless of normal appearance on diagnostic imaging.
4	All	Recommend removing a regional lymph node only if it is suspicious on diagnostic imaging and/or suspicious/positive for metastasis following cytologic assessment.
5	All	Other.

Sentinel lymph node mapping

16. Does the institution you are affiliated with use sentinel lymph node mapping in the head?

A. Yes
B. No

17. What type of sentinel lymph node mapping is performed at your institution?

____

18. How often is sentinel lymph node mapping successful in identification of the first draining node? (Please approximate in %)

____

19. Are you currently only removing the SLN or are you removing all draining nodes despite mapping prior to surgery for data collection?

A. Pulling all nodes despite mapping prior to surgery for data collection and histopathological examination
B. No, we are only removing the SLN for histopathological examination
C. Other

20. If only the SLN was removed and it was found to be metastatic on histopathology, do you recommend removal of the other lymphocenters or only adjunctive therapy (radiation of the lymph node basin/chemo)? **** This question was removed from analysis. The question and available answers did not appropriately match**

A. Yes
B. No

21.Does your institution have specific histopathological requirements after SLNs are removed? (i.e. bivalving the SLN through the hilum, breadloafing, use of IHC, or PCR analysis to detect mRNA associated metastatic differentiation)

____

22.Have you noted any associated complications that occur post-operatively after SLN mapping (i.e imaging associated complications)?

____

23.What is the approximate percent (%) of complications?

____

24. Please select 1 or more complications you have noted with lymphadenectomy in the neck at your institution:

A. Seroma – commonly >75% of the time
B. Seroma - approximately 50% of the time
C. Seroma - rarely <25%

D. Infection - commonly >75% of the time
E. Infection - approximately 50% of time
F. Infection - rarely <25%
G. Other

25. What other complications have you noted with lymphadenectomy in the neck at your institution?

____
